# Rapid isolation of mycoviral double-stranded RNA from *Botrytis cinerea *and *Saccharomyces cerevisiae*

**DOI:** 10.1186/1743-422X-8-38

**Published:** 2011-01-25

**Authors:** Antonio Castillo, Luis Cottet, Miguel Castro, Felipe Sepúlveda

**Affiliations:** 1 Laboratorio de Virología de Hongos, Departamento de Biología, Facultad de Química y Biología, Universidad de Santiago de Chile. Avenida Libertador Bernardo O'Higgins 3363, Estación Central, Santiago, Chile

## Abstract

**Background:**

In most of the infected fungi, the mycoviruses are latent or cryptic, the infected fungus does not show disease symptoms, and it is phenotypically identical to a non-infected strain of the same species. Because of these properties, the initial stage in the search for fungi infected with mycoviruses is the detection of their viral genome, which in most of the described cases corresponds to double-stranded RNA (dsRNA). So to analyze a large number of fungal isolates it is necessary to have a simple and rapid method to detect dsRNA.

**Results:**

A rapid method to isolate dsRNA from a virus-infected filamentous fungus, *Botrytis cinerea*, and from a killer strain of *Saccharomyces cerevisiae *using commercial minicolumns packed with CF11 cellulose was developed. In addition to being a rapid method, it allows to use small quantities of yeasts or mycelium as starting material, being obtained sufficient dsRNA quantity that can later be analyzed by agarose gel electrophoresis, treated with enzymes for its partial characterization, amplified by RT-PCR and cloned in appropriate vectors for further sequencing.

**Conclusions:**

The method yields high quality dsRNA, free from DNA and ssRNA. The use of nucleases to degrade the DNA or the ssRNA is not required, and it can be used to isolate dsRNA from any type of fungi or any biological sample that contains dsRNA.

## Background

Mycoviruses or fungal viruses have properties that differentiate them from viruses that infect animals, plants and bacteria [1-4]; they do not infect intact cells and are transmitted vertically by intracellular routes (meiosis and mitosis) and horizontally by anastomosis of compatible hyphae or through sexual mating of yeast cells. Mycoviruses may also be latent and/or cryptic, since in most cases the infected fungus does not show disease symptoms and is phenotypically identical to a non-infected strain of the same species. Due to these peculiarities, the initial stage in the search for infected fungi with mycoviruses is the detection of their viral genome, which in most of the described cases corresponds to dsRNA [1-4]. Although the number of ssRNA viruses described so far, such as the F and X viruses of *Botrytis cinerea *[4-6], has increased considerably, dsRNA continues to be the more predominant mycoviral genome. Therefore, to analyze a large number of fungal isolates it is necessary to have a rapid method that allows the isolation and partial characterization of viral dsRNA using small amounts of mycelia or yeast cells as starting material.

Some of the main and general methods described until now to isolate dsRNA molecules are: total nucleic acid isolation and further enzymatic digestion of the DNA and ssRNA [7]; phenol acid extraction (pH 4.5) in the presence of ammonium sulphate [8]; boiling of the fungal sample in the presence of a high salt concentration buffer [9], and use of CF11 cellulose, a chromatographic resin that allows the selective separation of dsRNA from DNA and ssRNA, using 16% ethanol in the elution buffer [10-13]. All of the former methods require a considerable quantity of initial sample to obtain sufficient dsRNA for its later characterization, so it is very difficult to analyze a large number of fungal isolates with these techniques.

Of the previous methodologies, the most widely used one is chromatographic separation on CF11-cellulose, since it allows getting dsRNA free of ssRNA, rRNA or tRNA, without further treatment.

In this paper we describe a rapid method for isolating dsRNA from a filamentous fungus, *Botrytis cinerea*, and a yeast, *Saccharomyces cerevisiae*. Besides being a rapid method, it allows the use of small amounts of yeasts or micelia as initial material to obtain a sufficient quantity of dsRNA that can later be analyzed by electrophoresis in agarose gel, quantified by densitometric analysis, and treated with enzymes for their partial characterization. The method allows getting high quality dsRNA, free of DNA and ssRNA, and it can be applied to isolate dsRNA from any type of fungus or any biological sample that contains dsRNA.

## Methods

### Fungal strains and culture conditions

*Botrytis cinerea *strains CCg378, THg324, and SUg275 were grown at 20°C for 7-10 days in 50 mL of liquid culture medium containing 1.5% (w/v) malt extract and 0.75% (w/v) yeast extract (Merck, Darmstadt, Germany). *S. cerevisiae *1743 (kindly provided by Dr. Reed B. Wickner) [14] was grown for 16-20 hours in 50 mL of liquid YPD medium containing 1.0% (w/v) yeast extract, 2.0% (w/v) peptone and 2.0% (w/v) glucose. In both cases the culture media were sterilized by autoclaving at 121°C for 20 min.

### dsRNA purification

**Figure 1:**
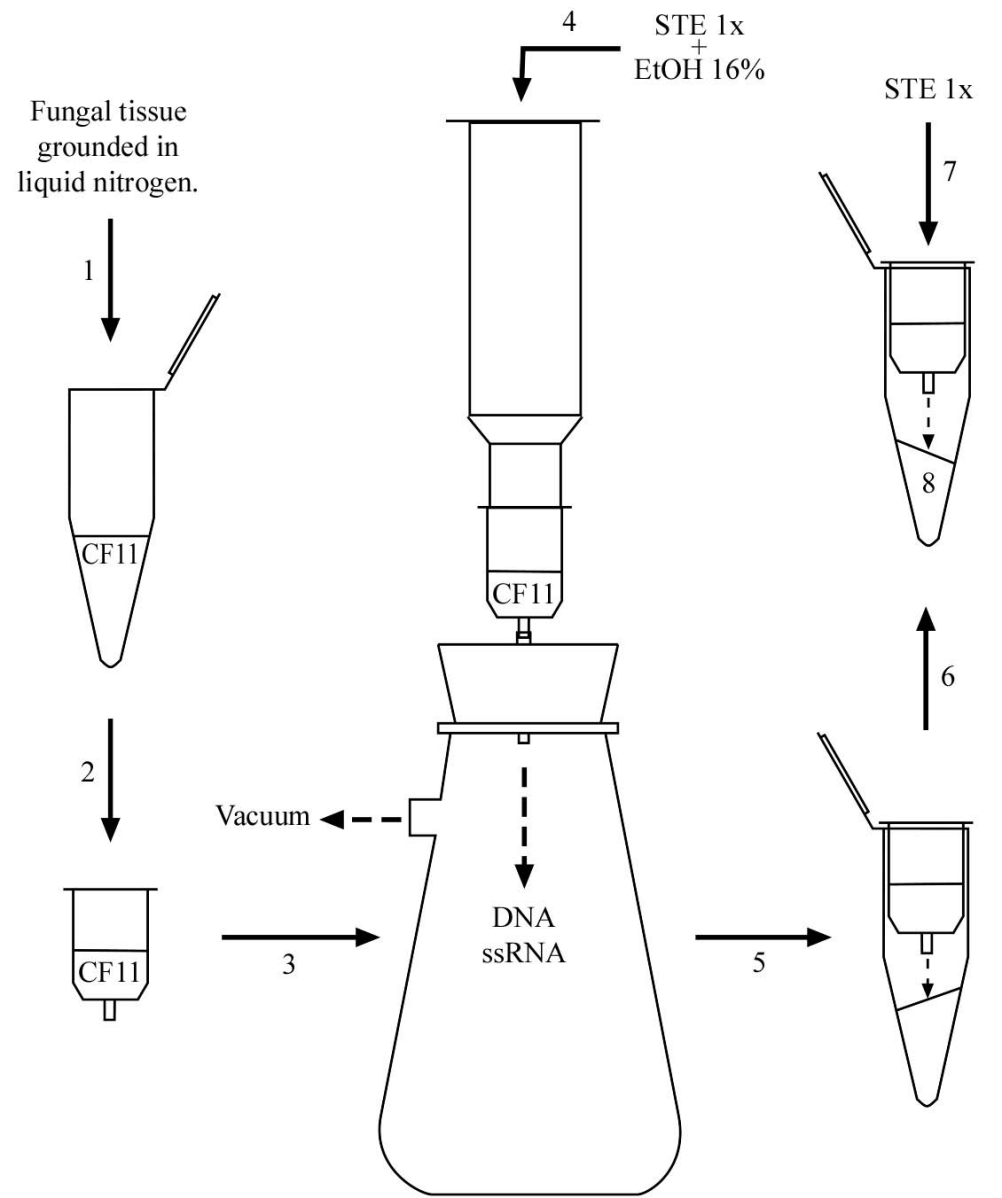
**Schematic representation of the steps that should be carried out to purify dsRNA**. For details of the technique, see dsRNA purification in Methods.

For the three *B. cinerea *strains the mycelia were manually separated from culture media with forceps and excess moisture was removed by pressing between paper towels. *S. cerevisiae *1743 yeast cells were sedimented by centrifugation and excess moisture was removed by incubation at 60°C for 10-15 min. The following steps are the same for both fungi and are represented schematically in Figure 1.

**(1) **Both the fungal mycelium and yeast cell pellet (3-5 g wet weight) were frozen in liquid nitrogen and ground to a fine powder with mortar and pestle. The powder was resuspended in 5 mL of STE 1X buffer (25 mM Tris-HCl pH 7.5, 50 mM NaCl and 0.5 M EDTA) containing 50 μL of β-mercaptoethanol. Then one volume of phenol:chloroform:isoamyl alcohol solution (25:24:1) was added. The mixture was stirred for 10 minutes on ice and was then centrifuged at 10,000 × g for 15 minutes. The aqueous phase was transferred to a sterile tube, ethanol was added to a final concentration of 16% (v/v) and the mixture was centrifuged for 15 minutes at 10,000 × g. The supernatant containing the total nucleic acids was recovered, discarding the pellet containing only a fraction of DNA and proteins.

**(2 & 3) **CF11 cellulose Whatman (0.2 g) prewashed with STE buffer containing 16% (v/v) ethanol and 2% (v/v) β-mercaptoethanol was added to a previously used commercial minicolumn without its original resin (Promega Wizard Plus Midipreps or Qiagen QIAprep empty Minicolumns). The minicolumn was coupled to a 5 mL syringe and mounted in a vacuum system.

**(4)**The supernatant (recovered in step 1), containing the total nucleic acids, was loaded in the minicolumn and eluted under vacuum, then 5 mL of STE buffer containing 16% (v/v) ethanol were immediately added to completely elute the DNA and ssRNA.

**(5) **The minicolumn was coupled to a 1.5 mL microcentrifuge tube and centrifuged for 30 s at 10,000 × *g *to eliminate the residual washed buffer.

**(6 & 7) **In order to recover dsRNAs bound to the resin, the minicolumn was coupled to a new microcentrifuge tube and 200 μL of STE buffer without ethanol were added over the CF11 cellulose contained in the minicolumn. The dsRNA was eluted by centrifugation for 2 min at 10,000 × g. Each recovered sample was added to 0.2 g of CF11 cellulose prewashed with STE buffer containing 16% (v/v) ethanol and 2% (v/v) β-mercaptoethanol, and steps 3 to 10 were repeated.

**(8) **Double-stranded RNA was precipitated overnight with 2 volumes of absolute ethanol at -20°C.

**(9 & 10) **After centrifugation for 15 min at 10,000 × g, the pellet containing the dsRNA was dried and resuspended in 10 μL of sterile triple-distilled water for its further analysis. The electrophoretic characterization of dsRNA was performed in 0.8% (w/v) agarose gel using TAE as running buffer (2 μL of dsRNA sample is sufficient to visualize bands of regular intensity in gel). The gel was subsequently stained by incubation in 0.5 μg/mL of ethidium bromide.

### Nucleic acid analysis

The electrophoretic and RNase A treatment conditions were as described by Castro et al. [13].

### Densitometric analysis

With the images of the gels, densitometry curves of the bands were processed with the aid of specific MediaCybernetics, Gel-Pro Analyzer Version 6.0 software.

### Molecular cloning of the 2.2 kpb dsRNA from B. cinerea CCg378

The dsRNAs of *B. cinerea *CCg378 were purified by CF11 cellulose chromatography and separated by agarose gel electrophoresis. Then, the 2.2 kbp band was cut-out of the gel and the dsRNA molecules were eluted putting the agarose piece in a eppendorf tube with triple-distilled water and incubating it to 4°C overnight. The agarose-free dsRNAs molecules were concentrated by ethanol precipitation. The obtaining of the cDNA by reverse transcription, the cDNA amplifying by PCR and cloning of the cDNA fragments in pGEM-T easy vector (Promega) were done essentially as described by Darissa et al. [15], using the single-primer amplification technique (SPAT). For the cDNA amplification we used the Go *Ta*q DNA polymerase with the colorless buffer (Promega).

## Results and Discussion

### Electrophoretic analysis of nucleic acids obtained from Botrytis cinerea and Saccharomyces cerevisiae

**Figure 2:**
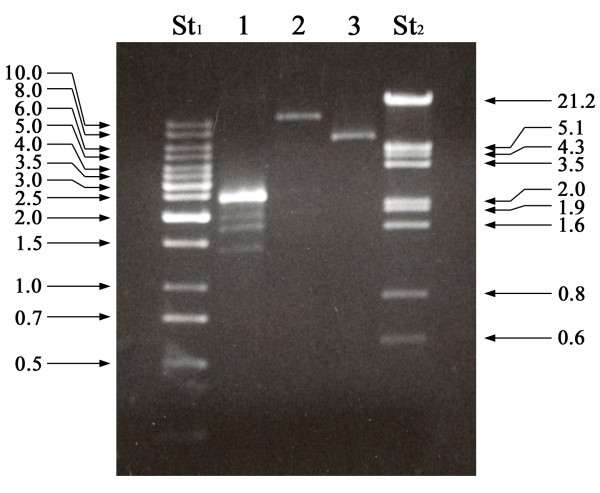
**Agarose gel electrophoresis of dsRNA from *Botrytis cinerea *wild-type strains**. Lane St_1_, O'GeneRuler™ 1 kb DNA Ladder, Fermentas; lanes 1, 2 and 3, dsRNA from *B. cinerea *CCg378, THg324 and SUg275 wild-type strains; lane St_2_, Lambda DNA/*Eco*RI + *Hin*dIII marker. The numbers on the left and right side indicate molecular sizes expressed in kilobase pairs (kbp).

The electrophoretic analysis of the CF11 cellulose column fractions, obtained after elution of the total nucleic acids using STE buffer containing 16% ethanol, revealed the presence of bands corresponding mainly to DNA and ssRNA (not shown). The dsRNAs retained by the CF11 cellulose resin were eluted with STE buffer without ethanol (Figure 2) and their chemical nature was demonstrated by their resistance to digestion with RNase A in a high ionic strength buffer. The dsRNAs obtained from different *Botrytis cinerea *strains are shown in Figure 2. The electrophoretic profile of the CCg378 strain revealed the presence of four dsRNA bands with approximate molecular sizes of 2.2, 1.95, 1.75 and 1.4 kilobase pairs (kbp) (Figure 2, lane 1), whereas the THg324 and SUg275 strains contained only one dsRNA molecule of about 12.0 and 7.5 kbp, respectively (Figure 2, lanes 2 and 3).

According to their molecular size, the 2.2-kbp dsRNAs of *B. cinerea *CCg378 may correspond to or be part of the genome of a partitivirus [16], whereas those of the THg324 and SUg275 strains may correspond to the genome of members of the *Hypoviridae *and *Totiviridae *families, respectively [17,18].

**Figure 3:**
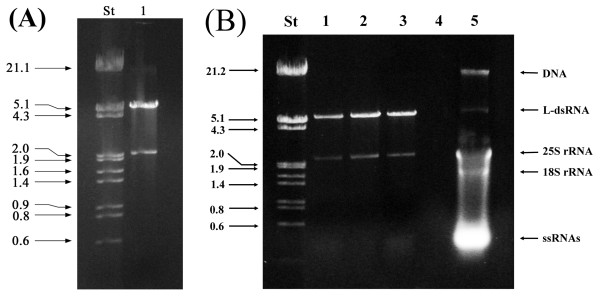
**Agarose gel electrophoresis of dsRNA from *Saccharomyces cerevisiae *1743**. **(A) **Lane St, Lambda DNA/*Eco*RI + *Hin*dIII marker; lane 1, dsRNAs from *Saccharomyces cerevisiae *1743. The numbers on the left side indicate molecular sizes expressed in kilobase pairs (kbp). **(B) **Lane St, Lambda DNA/*Eco*RI + *Hin*dIII marker; lanes 1, 2 and 3, different samples of dsRNA from *Saccharomyces cerevisiae *1743 (for details see *Binding capacity of dsRNA molecules by CF11 cellulose *in Results and Discussion); lane 4, empty; lane 5, nucleic acids of *S. cerevisiae *1743 eluted with STE buffer containing 16% (v/v) ethanol. The column was loaded with 4 mg of total nucleic acids. The numbers on the left side indicate molecular sizes expressed in kilobase pairs (kbp).

The purified dsRNAs of *S. cerevisiae *1743 are shown in Figure 3A. The 4.6 kbp L-dsRNA of the L-A virus and its satellite 1.8-kbp M-dsRNA are clearly noticeable (Figure 3A, lane 1).

### Binding capacity of dsRNA molecules by CF11 cellulose

In order to determine the binding capacity of dsRNA by the CF11 cellulose resin, different amounts of total nucleic acids were loaded in the minicolumns until the binding sites of the resin were saturated with dsRNA molecules. To achieve this we worked with three parallel experiments using the total nucleic acid preparation of *S. cerevisiae *1743 [14]. Two, four and six milligrams of total nucleic acids were loaded into separate columns, each containing 0.2 g of CF11 cellulose. The electrophoretic profile of nucleic acids eluted with STE buffer containing 16% (v/v) ethanol (see stage 4 of methods) revealed that when the amount of total nucleic acids loaded on the column was approximately 4 mg, dsRNA bands were also seen in addition to the bands corresponding to DNA and ssRNAs (Figure 3B, lane 5). Three sharp bands that correspond to the genomic DNA, 25S rRNA and 18S rRNA from *S. cerevisiae *1743 were seen clearly in the gel (Figure 3B, lane 5). Furthermore, it was possible to visualize the band corresponding to the L-dsRNA, but it was not possible to see the band of the M-dsRNA, since it migrates at the same speed that 25S rRNA and therefore both bands overlap in the gel (Figure 3B, lane 5). These results indicate that a portion of the dsRNA molecules contained in the total nucleic acids was not binding to the resin, which was saturated without leaving binding sites available. Also, it was possible to see clearly that the amounts of dsRNA obtained from the minicolumn loaded with four and six milligrams of total nucleic acids are equivalent (Figure 3B, lanes 2 and 3), confirming that the resin was saturated with the dsRNAs contained in 4 mg of total nucleic acids. Considering this, for this particular experiment 4 mg of total nucleic acids would be a sufficient initial amount to ensure an adequate yield in relation to the amount of CF11 cellullose (0.2 g of resin) packed in the minicolumn.

The initial 5 g cell pellet contained approximately 14 mg of total nucleic acids. Therefore, roughly 1.5 to 2.0 g wet weight of cells would be enough as starting material for a column. Alternatively, 3 columns can be loaded with the total nucleic acids obtained from the 5 g wet weight of fungal cells.

Under these conditions, the amount of total dsRNA recovered from a minicolumn was approximately 4 μg, since of the 10 μL obtained, only 2 μL were loaded in each lane of the gel. This amount was enough to correctly visualize the bands in the gel after staining with ethidium bromide (Figure 3B, lanes 1, 2 and 3), since in the three lanes the bands corresponding to the L and M dsRNA from *S. cerevisiae *1743, were clearly visualized. According to the densitometric analysis, the amount of dsRNA corresponding to each band in the gel was approximately 600 and 200 ng for the L and M dsRNA, respectively (Figure 3B, lane 2).

### Molecular cloning of the 2.2 kbp dsRNA from B. cinerea CCg378

**Figure 4:**
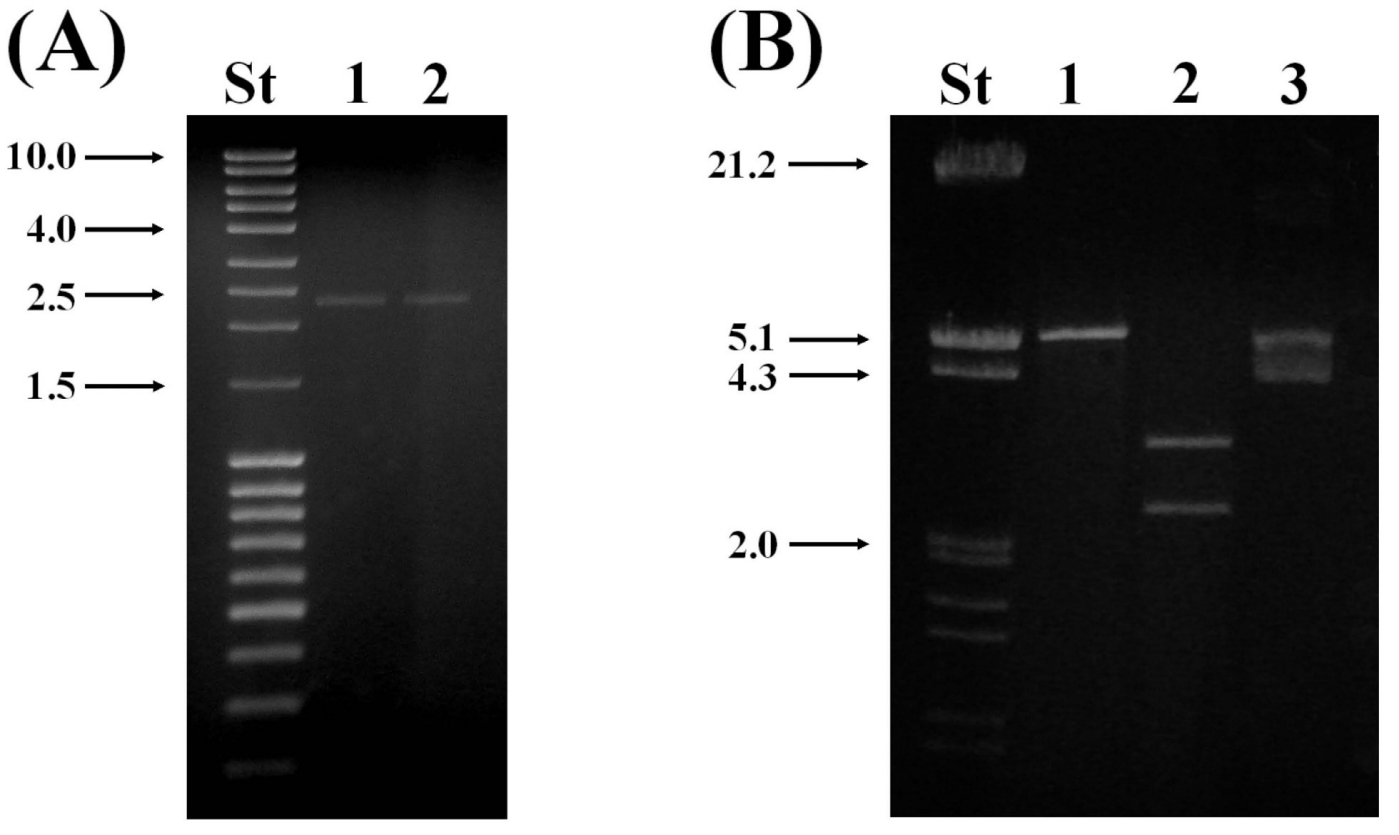
**Agarose gel electrophoresis of the cDNA obtained by the single-primer amplification technique**. ****(A) ****Lane St, MassRuler™ DNA Ladder 80-10,000 bp (Fermentas); lanes 1 and 2, PCR fragment corresponding to cDNA obtained from 2.2 kbp dsRNA from *B. cinerea *CCg378. The numbers on the left side indicate molecular sizes expressed in kilobase pairs (kbp). **(B) **Analysis of the digestion patterns with restriction enzymes of the recombinant plasmid (pGEM-T easy + cDNA) containing as insert the cDNA shown in (A). Lane St, Lambda DNA/*Eco*RI + *Hin*dIII marker; lane 1, recombinant plasmid treated with *Nco*I; lane 2, recombinant plasmid treated with *Nco*I and *Spe*I; lane 3, recombinant plasmid without treatment. The numbers on the left side indicate molecular sizes expressed in kilobase pairs (kbp).

To test the integrity and applicability of the dsRNA molecules purified by CF11 cellulose chromatography, the 2.2 kbp dsRNA band of *B. cinerea *CCg378 was eluted from the agarose gel, and after ligation of a synthetic oligonucleotide in their 3' ends, the cDNA was obtained by reverse transcription and amplified by PCR [15]. The obtained PCR fragment of about 2.2 kbp is shown in Figure 4A, lanes 1 and 2. Later, the cDNA was cloned in pGEM-T easy vector and the recombinant plasmid was characterized with restriction enzymes (Figure 4B). In Figure 4B, lane 1, the recombinant plasmid of about 5.2 kbp linearized with NcoI is shown. Treatment with NcoI and SpeI generated two bands, a corresponding to the cDNA of about 2.2 kbp and the other to the linear pGEM-T easy vector of about 3.0 kbp (Figure 4B, lane 2). Therefore, the obtention of the full-length cDNA corresponding to the 2.2 kbp dsRNA from *B. cinerea *CCg378, is a confirmation that the dsRNA molecules isolated using the methodology described in this work are obtained chemically intact and can be used for other applications, such as cDNA preparation, PCR, and molecular cloning.

The dsRNA purification technique presented in this paper is very fast and very easy to perform. The results show clearly that from small samples (mycelia or yeast cells) it was possible to obtain dsRNAs free from DNA and ssRNA, and in sufficient amount for their preliminary characterization. Therefore, using this methodology it should be possible to analyze simultaneously a large number of fungal strains to detect the presence of mycoviral dsRNA. These data show that neither the origin, the source, the size, or the number of dsRNA segments are impediments to obtain dsRNA free from DNA and ssRNA.

A similar methodology has been described to isolate dsRNA from the fungus *Paecilomyces*, but it requires treatment with DNase I to remove the DNA present in the dsRNA preparations [19]. Another technique that uses guanidinium thiocyanate as the main reagent to isolate dsRNA of *Uncinula necator*, requires that the dsRNA samples be treated with RNase A to eliminate ssRNAs that are visualized as smears in the lanes where the obtained dsRNA samples had been loaded [20]. More recently a technique that uses polyvinylpolypyrrolidone instead of phenol-chloroform has been described [21]. This procedure is very similar to that described in this paper, and the results are equivalent in terms of the electrophoretic quality of the dsRNA. However, it is not possible to make a quantitative comparison of both methods, since the authors of that paper do not quantify the dsRNA obtained.

Two aspects that have been improved in the methodology described in the present paper are the required time and the amounts of reagents used. The original technique of CF11cellulose chromatography [10-13] requires that the sample be incubated overnight with the resin, followed by two chromatographic cycles to obtain high purity dsRNA after three days of work. In the case of the minicolumns described in this paper, the sample is loaded directly in the column, eliminating the incubation time with the resin, and only about 20 minutes of elution are needed to obtain high purity dsRNA.

It is worth noting that in the original method of chromatography on cellulose CF11, 15 to 20 grams of mycelium or yeast cells are needed, and the total nucleic acid extract obtained is used wholly to make a column of about 20 mL, while in the case of a minicolumn only 2 mL of total nucleic acid extraction are required, thus allowing running two chromatographic experiments in parallel, using only about 2 g of mycelia or yeast cells as starting material.

The most critical aspects of the technique are related to cellular breaking and the degree of hydration of the chromatographic resin during the whole procedure. In order for the breaking of the mycelia or yeast cells to be more efficient it is advisable to withdraw most of the water from this starting material, as described in methods section. With the addition of liquid nitrogen, aqueous crystals would be formed if the samples have too much moisture. Under these conditions the cell breaking would be very difficult and the final yield would decrease significantly. In the whole purification process of dsRNA the resin (CF11 cellulose) must remain hydrated. If for some reason the resin becomes dehydrated, the yield will decrease remarkably and in extreme cases may result in no production of dsRNAs.

## Conclusions

We developed a very simple and rapid method to isolate dsRNA from fungi, using commercial minicolumns packed with CF11 cellulose. From small quantities of fungal cells as initial samples it was possible to obtain sufficient amount of chemically intact dsRNA for its partial characterization. The dsRNA obtained is electrophoretically free of other nucleic acids, such as DNA and ssRNA, and no additional treatment with nucleases was required. We consider that this methodology may be used to isolate dsRNA from any type of fungi and also can be applied to isolate genomic RNA from dsRNA viruses that infect cells of higher eukaryotes, since the phenol-chloroform extraction would help to eliminate capsid proteins. We think that this method may be used to isolate dsRNA from any biological sample that contains dsRNA and that the dsRNA obtained can be used for further downstream applications.

## Competing interests

The authors declare that they have no competing interests.

## Authors' contributions

AC and MC designed the study, participated in the characterization of the dsRNA, performed the densitometric analysis, performed the molecular cloning of the 2.2 kbp dsRNA from *B. cinerea *CCg378 and co-wrote the manuscript; LC and FS carried out the isolation, purification and characterization of dsRNA from *Saccharomyces cerevisiae *and *Botrytis cinerea*. All the authors read and approved the final manuscript.
